# The 90‐Day Survival Threshold: A Pivotal Determinant of Long‐Term Prognosis in HBV‐ACLF Patients – Insights from a Prospective Longitudinal Cohort Study

**DOI:** 10.1002/advs.202304381

**Published:** 2024-02-21

**Authors:** Lanlan Xiao, Jiajia Chen, Shuai Zhao, Wenxin Zhoudi, Keting He, Xiaohan Qian, Fen Zhang, Qiuhong Liu, Tan Li, Danhua Zhu, Xiaoxin Wu, Zhangya Pu, Jianrong Huang, Zhongyang Xie, Xiaowei Xu

**Affiliations:** ^1^ State Key Laboratory for Diagnosis and Treatment of Infectious Diseases National Clinical Research Center for Infectious Diseases National Medical Center for Infectious Diseases Collaborative Innovation Center for Diagnosis and Treatment of Infectious Diseases The First Affiliated Hospital Zhejiang University School of Medicine 79 Qingchun Rd. Hangzhou City 310003 China

**Keywords:** acute‐on‐chronic liver failure, HBeAg seroconversion, hepatitis B virus, liver fibrosis, long term prognosis, virological responses

## Abstract

This work aims to explore the long‐term prognosis of hepatitis B virus‐related acute‐on‐chronic liver failure (HBV‐ACLF). In this prospective study, eligible inpatients with HBV‐ACLF are enrolled and followed up from December 2012 to February 2023, for clinical events, laboratory tests at least every 6 months. Overall, the survival rates at 28 days, 90 days, 1 year, 5 years, and 8 years are 64.7%, 48.8%, 46.1%, 43.8%, and 42.2%, respectively. Among the 8‐year mortality and liver transplant cases, ACLF survivors (who survived over 90 days) accounted for 7.8% (9/115). Among 101 patients who survived for more than 90 days, 97.9% of patients achieve virologic response at 1 year. For HBeAg‐positive patients, the HBeAg seroconversion are 25.5%, 63.6%, and 76.9% at 1, 5, and 8 years, respectively. Alanine aminotransferase, aspartate aminotransferase, total bilirubin, INR, white blood cell count, and albumin levels gradually improve within the first year. Fibrosis biomarkers APRI, FIB‐4 and Chitinase‐3‐like protein 1 (CHI3L1) levels decreases within the first 5 years. The Cox proportional hazards regression reveal that high total bilirubin (HR = 1.008, *p* = 0.021) is the independent risk factor for 8‐year survival of ALCF survivors. The 90‐day period following of HBV‐ACLF represented a critical juncture for long‐term prognosis, revealing favorable outcomes beyond this timeframe.

## Graphical abstract

The 90‐day period following the occurrence of HBV‐ACLF is a critical time point for long‐term prognosis of HBV‐ACLF patients, with favorable outcomes of survival rates, laboratory indicators, virological, and serologic responses and values of liver fibrosis indicators observed beyond this timeframe. Moreover, the high levels of total bilirubin might be the underlying risk factor for 8‐year survival of ALCF patients.

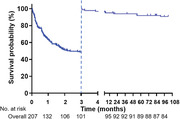

## 1 Introduction

Hepatitis B virus (HBV)‐related acute‐on‐chronic liver failure (HBV‐ACLF) is a complex syndrome that occurs in patients with chronic hepatitis B (CHB), and it is associated with a high short‐term mortality rate of 50%–90% unless patients promptly undergo liver transplantation (LT). HBV‐ACLF is the most common type of ACLF in the Asia‐Pacific and African regions and is mainly caused by the acute severe exacerbation of CHB,^[^ ^1^ ^]^ usually induced by hepatitis B activation (36%) and bacterial infection (28%).^[^ ^1^, ^2^ ^]^ In contrast, ACLF in the Western countries is often induced by bacterial infection (33%) and active alcoholism (24%) on the basis of alcoholic cirrhosis and Hepatitis C cirrhosis.^[^ ^3^ ^]^ Compared with non‐HBV‐ACLF, HBV‐ACLF shows higher short‐term mortality, more prominent liver failure and coagulation failure.^[^ ^3^, ^4^ ^]^

While many studies have primarily focused on short‐term clinical manifestations and prognosis due to the high short‐term mortality rate of ALCF, understanding the long‐term prognosis of HBV‐ACLF patients is equally important, especially for its decisive role in the long‐term management of HBV‐ACLF patients. We hypothesize that the majority of deaths in HBV‐ACLF patients occur in the early stages of the disease, and patients who successfully overcome the acute phase have a favorable prognosis.

Furthermore, clinical trials have shown that long‐term regular antiviral treatment with nucleos(t)ide analogs can lead to the regression of fibrosis and cirrhosis in patients with chronic hepatitis B (CHB),^[^ ^5^, ^6^, ^7^ ^]^ and active antiviral treatment can improve productivity in HBV‐ACLF patients.^[^ ^8^ ^]^ However, currently, there is a lack of relevant research reports on whether patients with HBV‐ACLF experience improvements in liver cirrhosis and fibrosis after receiving long‐term antiviral treatment. Therefore, it is necessary to conduct a long‐term follow‐up study to explore the entire disease course, including long‐term mortality rates, adverse clinical events, and regression of advanced liver fibrosis/cirrhosis in patients with HBV‐ACLF.

## 2 Experimental Section

### 2.1 Study Design

This prospective cohort study was conducted between December 2012 and February 2023, at the First Affiliated Hospital of Zhejiang University (Hangzhou, China). Eligible hospitalized patients with HBV‐ACLF were screened between December 2012 and May 2015 and enrolled patients underwent at least 8 years follow‐up. The study protocol received approval from the Ethics Committee of the First Affiliated Hospital, Zhejiang University School of Medicine, and written informed consent was obtained from all patients involved in this study.

### 2.2 Patients

Patients were diagnosed with CHB according to the 2009 American Association for the Study of Liver Diseases (AASLD) guidelines.^[^ ^9^ ^]^ HBV‐ACLF was diagnosed according to the Asian Pacific Association for the Study of the Liver criteria (APASL 2009).^[^ ^10^ ^]^ The diagnosis of cirrhosis was established through a combination of previous liver biopsy, clinical and laboratory evidence of decompensation, endoscopic examination (esophageal and gastric varices), and radiological imaging demonstrating signs of portal hypertension and/or liver nodules. The exclusion criteria included: 1) age < 18 or > 80 years, 2) history of hepatocellular carcinoma (HCC) or other liver malignancies, 3) history of other tumors, 4) HIV infection, 5) survival time of 1 day following admission, and 6) presence of a severe comorbidity that could affect survival.

Patients with CHB and healthy subjects were enrolled as the control cohort of the ACLF population. Liver fibrosis in CHB patients was determined via liver biopsy (Metavir Fibrosis Stage: F0–F2).^[^ ^11^ ^]^ Healthy controls were defined as healthy people over 18 years old without viral hepatitis, liver cancer, alcoholic liver disease and other related liver diseases.

### 2.3 Treatment and Follow‐Up

**Figure 1:**
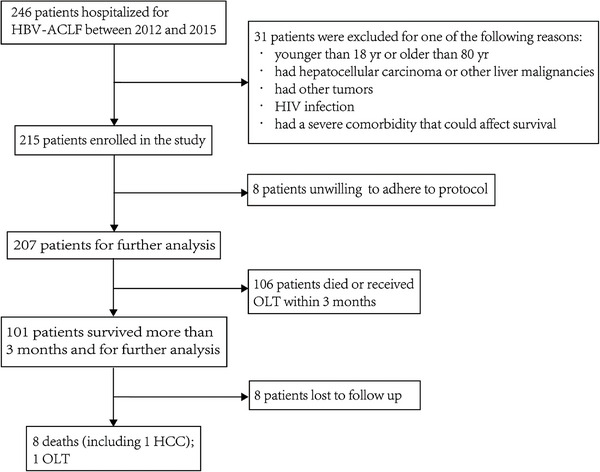
Flowchart of the study. OLT, orthotopic liver transplantation; HCC, hepatocellular carcinoma.

Eligible patients were invited to participate in this study, and those who survived for more than 90 days without undergoing liver transplantation (LT) (referred to as ACLF survivors) were included in the long‐term follow‐up (**Figure** **1**). Patients were evaluated at baseline and at weeks 1, 2, 3, 4, 8, 12, and 24, followed by assessments every 24 weeks until February 2023. The longest follow‐up duration was 408 weeks (Table S1, Supporting Information). Data on symptoms, physical status, complications (e.g., Gastrointestinal bleeding, HCC, etc.), LT and death event were recorded. Laboratory tests were conducted at each visit, including biochemistry, blood routine, coagulation, HBV DNA, HBV serological markers (HBV DNA, hepatitis B surface antigen (HBsAg), hepatitis B e antigen (HBeAg) and anti‐HBe) and alpha‐fetoprotein (AFP). The severity of ACLF was classified based on the Chinese Group on the Study of Severe Hepatitis (COSSH) criteria.^[^ ^4^ ^]^ Pre‐ALCF is defined as a condition where the severity of the ACLF meets the APASL 2009 criteria but does not meet the grade 1 of the COSSH criteria. HBV DNA levels was quantified using a real‐time PCR assay with a lower limit of quantification of 30 IU mL^−1^. Virological response was defined as an undetectable level of HBV DNA. HBV reactivation was One of the following: 1) a ≥ 100‐fold increase in the HBV DNA load from baseline levels; 2) the reappearance of HBV DNA to a level of 100 IU mL^−1^ if the baseline level is undetectable.^[^ ^12^ ^]^ The loss of HBeAg and the production of anti‐HBe were referred to as HBeAg seroconversion.^[^ ^13^ ^]^ The loss of HBeAg and the production of anti‐HBe were referred to as HBeAg seroconversion. To further analyze the indicators of liver fibrosis, we enrolled 24 patients with CHB and 14 healthy subjects. The demographics of the healthy controls and CHB patients are presented in Table S2, Supporting Information. Chitinase‐3‐like protein 1 (CHI3L1), is a highly liver‐enriched protein and a driving force of liver fibrosis, serves as a novel noninvasive liver fibrosis biomarker that can accurately stages different stages of liver fibrosis^[^ ^14^ ^]^ according to the Metavir Fibrosis Stage^[^ ^11^ ^]^ (F0–F4) classification. The aspartate aminotransferase (AST)‐to‐platelet ratio (APRI) and fibrosis‐4 (FIB‐4), two commonly used fibrosis indicators,^[^ ^15^ ^]^ along with CHI3L1^[^ ^16^, ^17^ ^]^ were used to evaluate liver fibrosis.

During hospitalization, all patients received standard medical therapy.^[^ ^18^, ^19^ ^]^ At each clinic visit, the investigators provided guidance to patients on medication and nucleos(t)ide analogs based on their individual condition. Continuous medication treatment with nucleos(t)ide analogs was administered to all patients.

### 2.4 Measurement of Serum CHI3L1

Blood samples were obtained at admission and at each follow‐up time point, and centrifuged at 4 °C for 10 min at 3500 rpm. The serum samples were stored at −80 °C. CHI3L1 is a glycoprotein with high liver specificity and demonstrates high diagnostic sensitivity and specificity for the diagnosis of liver fibrosis.^[^ ^16^, ^17^ ^]^ CHI3L1 levels were assayed using an enzyme‐linked immunosorbent assay (ELISA) with a human CHI3L1 ELISA kit (Abcam).

### 2.5 Outcome Assessment

The primary outcome assessed was survival rate, which not included death and LT. The secondary outcomes included the following: 1) occurrence of new liver‐related events (hepatic encephalopathy,^[^ ^20^ ^]^ gastrointestinal bleeding,^[^ ^1^ ^]^ hepatocellular carcinoma [HCC]^[^ ^21^ ^]^ etc.); 2) virological and serologic responses; 3) changes in the values of liver fibrosis indicators.

### 2.6 Statistical Analysis

Categorical data were reported as numbers (percentages) and compared using either the chi‐squared test or Fisher's exact tests. Continuous data were presented as mean ± standard deviation, and categorical data were presented as number (percentages). For normally distributed variables, Student's *t*‐test was used for comparisons, while the Wilcoxon rank‐sum test was employed for non‐normal continuous variables. Survival rates were assessed utilizing the Kaplan‐Meier method or Landmark analysis. Multivariate survival analysis with Cox proportional hazards regression model was conducted to identify the risk factors of long‐term prognosis. To obtain the cut‐off value of underlying factor, the area under the curve of the receiver operating characteristic (AUROC) were calculated. Statistical analyses were conducted using SPSS (version 24.0, IBM, Armonk, NY) and a *p*‐value < 0.05 (two‐sided) was considered statistically significant.

## 3 Results

### 3.1 Patients

A total of 207 eligible participants were invited to enroll this study and were followed up (Figure 1). During the follow‐up period, 13 patients withdrew from the study due to factors such as long distance. Subsequently, telephone follow‐up was conducted on these patients, among them 8 patients were lost to follow‐up. The clinical and laboratory characteristics of the 207 patients are shown in **Table** **1**. Among them, 85.5% were men, with a mean age of 44.7 ± 10.6 years (range: 21–64 years). According to the COSSH criteria, there were 16 (7.7%) patients with pre‐ACLF grade, 111 (53.6%) with ACLF grade 1, 64 (30.9%) with ACLF grade 2, and 16 (7.7%) with ACLF grade 3. The most common complications observed were hepatic encephalopathy (55 [26.6%]), followed by overt ascites (30 [14.5%]), infection (20 [9.7%]), spontaneous bacterial peritonitis (19 [9.2%]), gastrointestinal hemorrhage (19 [9.2%]), and hepatorenal syndrome (17 [8.2%]). Evidence of cirrhosis at baseline was present in 92 (44.4%) patients, with 41 (44.6%) were ACLF survivors.

**Table 1:** Baseline clinical and laboratory characteristics.

Variable	Baseline (*n* = 207)	ACLF survivors (*n* = 101)	ACLF Nonsurvivors (*n* = 106)	HBV‐C (*n* = 92)	HBV‐NC (*n* = 115)
Age (years)	44.7 (10.6)	41.1 (8.9)	48.2 (10.9)***	48.0 (9.8)	42.1 (10.5)***
Male sex (no.)	177 (85.5)	85 (84.2)	92 (86.8)	79 (85.9)	98 (85.2)
Liver cirrhosis (no.)	92 (44.4)	41 (40.6)	51 (48.1)	/	/
Liver transplantation (no.)	18 (8.7)	1 (1.0)	17 (16.0)***	13 (14.1)	5 (4.3)*
Liver support system (no.)	70 (33.8)	27 (26.7)	43 (40.6)*	37 (40.2)	33 (28.7)
HBV‐DNA level (IU mL^−1^)					
≤200	7 (3.4)	3 (3.0)	4 (3.8)	4 (4.3)	3 (2.6)
200–2 × 104	63 (30.4)	35 (34.7)	28 (26.4)	30 (32.6)	33 (28.7)
2 × 104–2 × 106	81 (39.1)	39 (38.6)	42 (39.6)	32 (34.8)	49 (42.6)
≥2 × 106	56 (27.1)	24 (23.8)	32 (30.2)	26 (28.3)	30 (26.1)
Complications (no.)					
Overt ascites	30 (14.5)	7 (6.9)	23 (21.7)**	21 (22.8)	9 (7.8)**
HE	55 (26.6)	7 (6.9)	48 (45.3)***	34 (37.0)	21 (18.3)**
Spontaneous bacterial peritonitis	19 (9.2)	6 (5.9)	13 (12.3)	11 (12.0)	8 (7.0)
Gastrointestinal hemorrhage	19 (9.2)	6 (5.9)	13 (12.3)	13 (14.1)	6 (5.2)
Infection	20 (9.7)	9 (8.9)	11 (10.4)	11 (12.0)	9 (7.8)
Hepatorenal syndrome	17 (8.2)	1 (1.1)	16 (15.1)***	12 (13.0)	5 (4.3)
Laboratory data					
ALT (U L^−1^)	505.9 (536.7)	471.5 (558.7)	538.8 (515.4)	388.4 (445.6)	600.0 (584.7)**
AST (U L^−1^)	349.7 (365.8)	282.1 (345.4)	414.1 (374.6)**	304.6 (343.8)	385.7 (380.1)
Albumin (g dL^−1^)	30.9 (5.5)	31.3 (6.6)	30.6 (4.2)	30.8 (4.8)	31.0 (6.0)
TB(µmol L^−1^)	339.1 (181.9)	289.2 (95.3)	386.5 (227.2)***	338.8 (146.3)	339.3 (206.7)
Creatinine (µmol L^−1^)	66.5 (27.1)	62.2 (14.1)	70.7 (34.9)*	67.3 (25.5)	66.0 (28.5)
White blood cell count (109 L^−1^)	7.1 (3.3)	6.6 (3.0)	7.6 (3.5)*	6.9 (3.6)	7.3 (3.1)
Neutrophil count (109 L^−1^)	4.8 (2.8)	4.3 (2.4)	67.1 (11.8)*	4.7 (2.9)	5.0 (2.7)
Haemoglobin (g L^−1^)	130.0 (21.5)	131.6 (20.3)	128.0 (22.3)	126.1 (21.4)	132.9 (21.2)*
Platelet count (109 L^−1^)	116.8 (60.9)	121.7 (58.2)	112.1 (63.2)	102.0 (57.6)	128.6 (61.1)
Prothrombin time (s)	26.3 (9.4)	22.8 (6.2)	29.7 (10.7)***	25.8	26.8 (10.3)
INR	2.2 (0.8)	1.9 (0.5)	2.5 (0.9)***	2.2 (0.7)	2.3 (0.8)
Severity score					
MELD	23.0 (7.0)	20.5 (3.7)	24.7 (6.2)***	22.6 (5.6)	22.7 (5.5)
CLIF‐C ACLFs	39.3 (7.4)	35.2 (4.9)	16.4 (6.2)***	40.5 (8.0)	38.4 (6.7)*
HBV‐ACLF (COSSH criteria) (no.)					
pre‐ACLF	16 (7.7)	14 (87.5)	2 (12.5)**	10 (62.5)	6 (37.5)
ACLF grade 1	111 (53.6)	73 (65.8)	38 (34.2)***	43 (38.7)	68 (61.3)
ACLF grade 2	64 (30.9)	13 (20.3)	51 (79.7)***	27 (42.2)	37 (57.8)
ACLF grade 3	16 (7.7)	1 (6.3)	15 (93.75)***	12 (75.0)	4 (25.0)

Data are mean ± standard deviation, or number (percentage). *p* values (*<0.05, **<0.01, ***<0.001) for comparisons between ACLF survivors and nonsurvivors or between patients with HBV‐ACLF with and without cirrhosis. ACLF survivor, patients with HBV‐ACLF survived more than 90 days. HBV‐C, HBV‐ACLF with cirrhosis; HBV‐NC, HBV‐ACLF without cirrhosis; ALT, alanine aminotransferase; AST, Aspartate aminotransferase; TB, total bilirubin; INR, International normalized ratio; MELD, Model for end‐stage liver disease; ACLF, acute‐on‐chronic liver failure; CLIF‐C, Chronic Liver Failure Consortium; COSSH, Chinese Group on the Study of Severe Hepatitis B.

As expected, short‐term mortality was notably high, with an overall 28‐day survival rate of 64.7% (134/207) and a 90‐day survival rate of 48.8% (101/207). Patients who survived beyond 90 days were categorized as ACLF survivors, whereas those who died or underwent LT within 90 days were classified as non‐survivors. The baseline indicators of ACLF survivors were better than those of non‐survivors, as ACLF survivors had a lower frequency of hepatic encephalopathy (*p* < 0.001) and ACLF grades 2 or 3 (*p* < 0.001 for both). Additionally, AST (282.1 ± 345.4 U L^−1^ vs. 414.1 ± 374.6 U L^−1^, *p* = 0.009), total bilirubin (TB,289.2 ± 95.3 µmol L^−1^ vs. 386.5 ± 227.2 µmol L^−1^, *p* < 0.001), prothrombin time (22.8 ± 6.2 s vs. 26.3 ± 9.4 s, *p* < 0.001), and international normalized ratio, (INR, 1.9 ± 0.5 vs. 2.5 ± 0.9, *p* < 0.001) were also significantly lower in ACLF survivors.

### 3.2 Long‐term Prognosis

**Figure 2:**
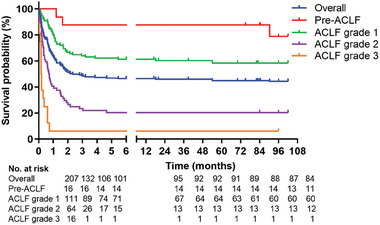
Long‐term survival curves for all HBV‐ACLF patients. ACLF, acute‐on‐chronic liver failure.

The 1‐, 2‐, 3‐, 5‐, and 8‐year survival rates were 46.1% (95/206), 45.1% (92/204), 45.1% (92/204), 43.8% (89/203), and 42.2% (84/199), respectively. The median survival time was 46.7 ± 43.5 months. The data clearly demonstrated that higher grade of ACLF were associated with increased mortality (**Figure** **2**).

The decline in survival rate was slow once the survival time exceeded 90 days. Among the 8‐year mortality and liver transplant cases, 92.2% (106/115) of them occurred within the first 90 days. Consequently, we conducted a long‐term survival analysis for the ACLF survivors (*n* = 101) who survived beyond 90 days. The relevant data analysis included thirteen patients who withdrew. The mean follow‐up time was of 86.51 ± 26.01 months (range 3.03–102), with a total of observation period of 728.17 person‐years.

During the follow‐up, eight patients died, and one received LT. For these patients, follow‐up was censored at the time of death or receiving LT (mean follow‐up of 26.31 ± 31.98 months, range: 3.03–90.34 months). The clinical characteristics of these patients (age, sex, MELD score, etc. Table S3, Supporting Information) did not significantly differ from those of patients who were still under observation at the end of the study. The main causes of death or receiving LT in 9 patients were liver failure (*n =* 4), gastrointestinal hemorrhage (*n =* 2), multiple organ failure (*n =* 1), sepsis (*n =* 1), and hepatocellular carcinoma (HCC; *n =* 1, **Table** **2**), respectively.

**Table 2:** Data on patients who died or underwent liver transplantation.

Sex	Age [years]	Cirrhosis	Survival time [months]	ACLF grade	State	Main causes of death or liver transplantation
Female	48	No	3.03	2	Death	Liver failure
Male	51	Yes	3.2	1	Death	Liver failure
Male	39	Yes	3.87	1	Liver transplantation	Liver failure
Male	39	No	4.67	1	Death	Multiple organ failure
Male	44	No	5.47	1	Death	Gastrointestinal hemorrhage
Male	47	No	18.23	1	Death	Sepsis
Male	59	Yes	54	1	Death	Hepatocellular carcinoma
Male	37	Yes	54	1	Death	Gastrointestinal hemorrhage
Male	49	Yes	90.34	1	Death	Liver failure

### 3.3 Changes in Laboratory Parameters

**Figure 3:**
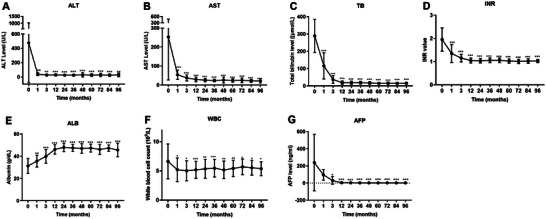
Changes in laboratory parameters in the survival patients (*n =* 101). ALT, alanine aminotransferase; AST, aspartate aminotransferase; TB, total bilirubin; INR, international normalized ratio; ALB, albumin; WBC, white blood cell count; AFP, alpha fetoprotein. *p* values (*<0.05, **<0.01, ***<0.001).

We conducted the long‐term monitoring of laboratory parameters in the ACLF survivors (**Figure** **3**). Notably, we observed significant decreases within the first 90 days in the alanine aminotransferase (ALT, 475.4 ± 556.1 U L^−1^ to 27.4 ± 11.9 U L^−1^), AST (253.1 ± 226.4 U L^−1^ to 37.6 ± 15.8 U L^−1^), TB (289.2 ± 95.3 U L^−1^ to 36.4 ± 22.4 U L^−1^), INR (2.0 ± 0.5 to 1.2 ± 0.2), white blood cell count (WBC, 6.6 ± 3.0 × 10^9^ to 5.0 ± 1.7 × 10^9^) and AFP (238.8 ± 329.2 ng mL^−1^ to 30.5 ± 44.1 ng mL^−1^). Additionally, the albumin gradually increased from 30.0 ± 6.7 to 48.0 ± 3.8 g L^−1^ within the first 2 year (Figure 3). During the following long term follow‐up period, the clinical features remained relatively stable.

### 3.4 Virological and Serologic Responses

**Figure 4:**
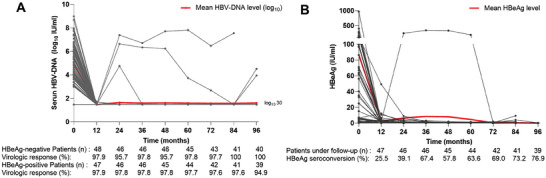
Virological and Serologic Responses. A: The change of HBV DNA level (log _10_ IU mL^−1^) and virological response (HBV DNA level <30 IU mL^−1^). B: The change of HBeAg level (IU mL^−1^) and HBeAg seroconversion (loss of HBeAg and development of antibodies to HBeAg).

Following HBV antiviral treatment, the HBV DNA level significantly decreased during the first year. At 1 year, both 97.9% of hepatitis B e antigen (HBeAg)‐negative and HBeAg‐positive patients achieved a virologic response (HBV DNA level <30 IU mL^−1^). Meanwhile, 97.8% HBeAg‐negative and 97.7% HBeAg‐positive patients achieved a virologic response at 5 years (**Figure** **4**). Meanwhile, HBeAg seroconversion (loss of HBeAg and development of antibodies to HBeAg) was observed in 25.5% (12/47), 63.6% (28/44), and 76.9% (30/39) of patients at 1, 5, and 8 years, respectively (Figure 4).

During the 8‐year follow up, five patients developed HBV reactivation. Two patients once again achieved virologic response after adjusting antiviral drugs. Two patients developed HBV reactivation in the eighth year of follow‐up, with HBV DNA levels of 3.28 × 10^61^ and 8.83 × 10^3^ IU mL^−1^, respectively. One patient maintained a high level of HBV‐DNA (3.01 × 10^6^ to 6.52 × 10^7^ IU mL^−1^) from the second to seventh year of follow‐up. All these five patients were currently alive. As shown in the Figure S1, Supporting Information, we found that the level of HBsAg in a small portion of patients decreased from the second year of follow‐up. However, none of the patients showed HBsAg seroconversion during the follow‐up period.

### 3.5 Changes in Fibrosis Index

**Figure 5:**
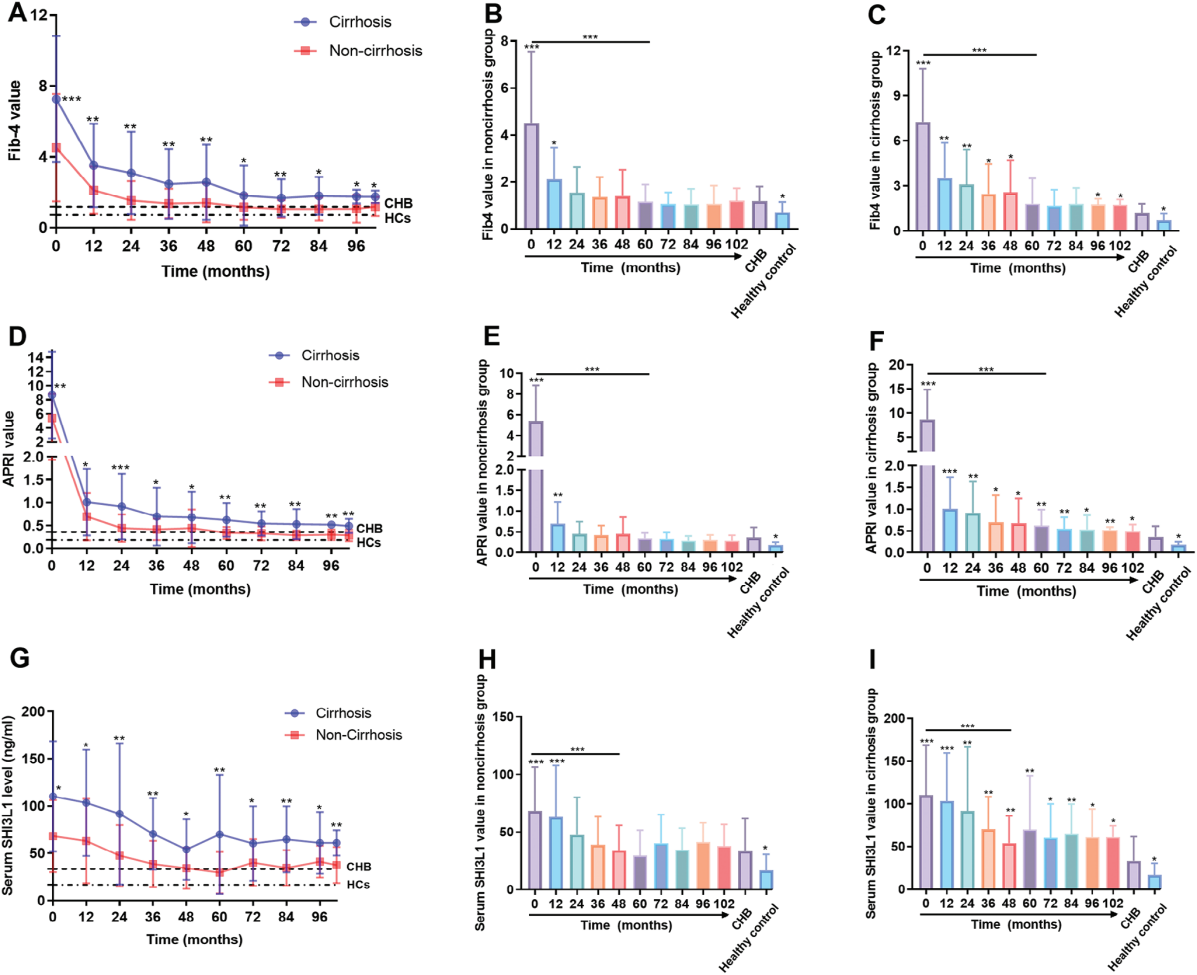
Changes in serum fibrosis markers in the survival patients (*n =* 101). In A, D, and G, *p*‐values were generated from comparisons between the cirrhosis and non‐cirrhosis groups. In B, C, E, F, H, and I, p‐values were generated from comparisons between the CHB and other groups. APRI, aspartate aminotransferase‐to‐platelet ratio; FIB‐4, Fibrosis‐4; CHI3L1, Chitinase‐3‐like protein. *p* values (*<0.05, **<0.01, ***<0.001).

Liver ultrasound, APRI, FIB‐4, and serum CHI3L1 were used to evaluate liver fibrosis. First, the B‐ultrasound results showed no transition from liver fibrosis to liver cirrhosis or vice versa. Second, in both the cirrhosis (*n =* 41) group and non‐cirrhosis (*n =* 60) group, APRI (baseline: 8.7 ± 6.1, 5.4 ± 3.4) and FIB‐4 (baseline: 7.3 ± 3.6, 4.5 ± 3.3) values gradually decreased in the first 5 years (APRI: 0.7 ± 0.4, 0.3 ± 0.1; FIB‐4: 1.8 ± 1.7, 1.2 ± 0.7, *p* < 0.001). Serum CHI3L1 (baseline: 110.3 ± 58.4, 68.3 ± 38.1 ng mL^−1^) levels also decreased gradually in the first 4 years (54.0 ± 32.0, 34.2 ± 21.7 ng mL^−1^, *p* < 0.001), followed by a stable trend. Throughout the long‐term follow‐up, patients with cirrhosis consistently presented higher levels of APRI, FIB‐4, and serum CHI3L1 compared to those without cirrhosis (*p* < 0.05, **Figure** **5**A,D,G). In the second year, the APRI, FIB‐4 and serum CHI3L1 levels in the non‐cirrhosis group decreased to levels similar with those of CHB patients (Figure 5B,E,H). Conversely, in cirrhosis group, the APRI, FIB‐4 and serum CHI3L1 levels were higher than those in CHB patients for most of the follow‐up period (Figure 5C,F,I).

### 3.6 Risk Factor for Long‐Term Prognosis

Multivariate survival analysis with Cox proportional hazards regression model was conducted on age, sex, cirrhosis, HBeAg, ALT, AST, TB, INR, APF, MELD, and COSSH grade for risk factors for long‐term survival of ACLF survivors (*n =* 101). The Cox proportional hazards regression analysis revealed that the high level of TB was (HR = 1.008 (95%CI 1.001‐1.015), *p* = 0.021) the independent risk factor for 8‐year survival of ACLF survivors (Table S4, Supporting Information). To obtain the cut off value of TB for 8‐year survival, then the AUROCs were calculated (Figure S2, Supporting Information). And the cut‐off value of TB level for predict the 8‐year survival were 354 µmol L^−1^. Then we made a landmark analysis for ACLF patients (*n* = 207) based on cut‐off value of TB level. The landmark analysis discriminated between different TB levels after 90 days of follow‐up (OR = 6.452 (95%CI 1.356‐30.69), *p* = 0.0175), while there was no difference before 90 days of follow‐up (OR = 1.028 (95%CI 0.7011‐1.507), *p* = 0.9192) (Figure S3, Supporting Information).

## 4 Discussion

Liver failure due to severe hepatitis B in China differs from that caused by alcohol and hepatitis C in the West.^[^ ^4^ ^]^ Although there have been numerous clinical studies on HBV‐ACLF, the long‐term follow‐up data remains limited. Therefore, there is an urgent need to explore the long‐term progression of HBV‐ACLF during extended antiviral treatment. This prospective cohort study with long‐term follow‐up examined the long‐term mortality, virological/serologic responses, and changes in liver fibrosis indicators in patients with HBV‐ACLF, and identified the independent risk factors for long‐term prognosis.

Consistent with previous reports and the COSSH study,^[^ ^4^ ^]^ the short‐term (28/90‐day) survival rates were notably low. The survival rates after 1, 2, 5, and 8 years were 46.1%, 45.1%, 45.1%, 43.8%, and 42.2%, respectively. Interestingly, the majority of deaths a in ACLF patients occurred within the first 90 days, accounting for 96.4% (106/110) of the 5‐year mortality. Beyond 90 days, the survival rates exhibited minimal changes up to 8 years. This viewpoint aligns with a study by Qin et al.,^[^ ^22^ ^]^ which reported that among 149 patients with HBV‐ACLF followed for 5 years, most deaths (75%, 111/149) occurred within the initial 90 days. Patients with HBV‐ACLF demonstrate distinct clinical characteristics that significantly differ from those of patients with non HBV‐ACLF.^[^ ^3^, ^4^, ^23^ ^]^ Similarly, Bruno et al. conducted a nationwide, prospective, 3‐year follow‐up study in Italy, where the cumulative incidence of failure (death or LT) increased over time, with rates of 28%, 53%, and 62% at 1, 2, and 3 years, respectively, in patients with ACLF. In their study, 40% of chronic liver disease cases were attributable to alcoholic hepatitis, and slightly more than half of the subjects were infected with hepatitis C virus. In contrast, all the patients in our study had CHB.^[^ ^24^ ^]^ Our findings highlighted the stark contrast in long‐term prognosis between HBV‐ACLF and non HBV‐ACLF, as the mortality rate among HBV‐ACLF patients who survive beyond 90 days is significantly lower. Moreover, we speculated that the 90‐day survival after HBV‐ACLF serves as a critical threshold for long‐term prognosis.

HBV infection is a major cause of ACLF in Asian countries, prompting the APASL guidelines to recommend immediate initiation of nucleos(t)ide analogs in HBV‐infected patients.^[^ ^10^ ^]^ Nucleos(t)ide analogs have been proven to effectively inhibit virus replication, improve histology and biochemistry, reduce the inflammatory response in CHB patients, and improve the short‐term prognosis of patients with HBV‐related ACLF.^[^ ^25^ ^]^ In our study, all patients were treated with nucleos(t)ide analogs throughout the study. Comparing the virological and serological responses between our HBV‐ACLF patients and the CHB patients from previous published trials^[^ ^7^ ^]^ who received nucleos(t)ide analog treatment yielded significant findings. A previous clinical trial^[^ ^13^ ^]^ showed that HBV DNA was undetectable by PCR assay in 67% of entecavir‐treated and 36% of lamivudine‐treated CHB patients respectively within the first year. Cumulative evidence confirmed that HBeAg seroconversion occurred in 21% of entecavir‐treated and 18% of lamivudine‐treated patients after 48 weeks of therapy. A 7‐year study of open‐label tenofovir disoproxil fumarate treatment for CHB patients reported that 99% of HBeAg‐negative patients and 97% of HBeAg‐positive patients achieved HBV DNA concentrations below 400 copies mL^−1^ at year 5. In addition, 40% of the patients achieved confirmed HBeAg seroconversion.^[^ ^7^ ^]^

Our study demonstrated that ACLF survivors had a higher virologic response rate at 1 year compared to CHB patients (97.9% vs 67%/36%, *p* < 0.05). However, no difference in virologic response rate was found between CHB patients and ACLF survivors in both the HBeAg‐negative (97.8% vs 98.3%) and HBeAg‐positive (97.7% vs 96.9%) groups at 5 years.^[^ ^7^ ^]^

The HBeAg seroconversion rate was significantly higher in ACLF patients than that of CHB patients at 2 years (39.1% vs 21%/18%, *p* < 0.05) and 5 years (63.6% vs 40%, *p* < 0.05) after treatment with nucleos(t)ide analogs. Based on the above results, we speculate that ACLF patients exhibit a stronger immune response and more efficient virus clearance in the early stage of antiviral treatment compared to CHB patients. A previous study^[^ ^26^ ^]^ revealed that HBV‐ACLF patients, in the absence of antiviral treatment, had lower serum HBV DNA load and serum HBsAg levels than CHB patients (*p* < 0.001, separately). This may also be attributed to the significant immune response following ACLF.

In individuals with CHB, continuous viral infection‐associated inflammation of the liver leads to the deposition of the extracellular matrix, resulting in liver fibrosis or cirrhosis, which can progress to end‐stage liver failure or HCC.^[^ ^27^ ^]^ Most cases of HCC occur in the presence of liver cirrhosis, with around half of all HCC cases associated with HBV infection.^[^ ^28^ ^]^ Increasing evidence also suggests that HBV‐related liver fibrosis or cirrhosis can regress histologically if HBV replication is suppressed.^[^ ^7^, ^29^ ^]^ However, the evolution of liver fibrosis after exposure to HBV‐ACLF remains unknown.

Although slowly, the value of fibrosis biomarkers FIB‐4, APRI and serum CHI3L1 in ACLF survivors of our study gradually declined in value during the fourth or fifth year of long‐term regular follow‐up and disease management (including antiviral therapy, prevention of complications, etc.), suggesting the potential for fibrosis regression in patients with HBV‐ACLF. Similarly, the APASL ACLF Research Consortium database^[^ ^30^ ^]^ reported reversibility in ≈70% ACLF survivors (who survived 90 days), with sustained improvements in enhanced hepatic reserve, reversal of fibrosis, and decreased portal pressure for at least 1 year.^[^ ^1^ ^]^ However, Zhu et al.^[^ ^31^ ^]^ reported cumulative progression rates of cirrhosis in 78 long‐term survivors of HBV ACLF at 12, 24, and 36 months as 37.9%, 58.4%, and 68.7, respectively. This disparity may be attributed to differences in disease management.

In this study, the fibrosis biomarkers show an extremely high value in the early days of ACLF. It is well‐established that acute liver injury, such as viral hepatitis, can induce the activation of hepatic stellate cells and liver macrophages,^[^ ^32^ ^]^ leading to an increased expression of CHI3L1,^[^ ^33^ ^]^ lifted AST/ALT levels, and decreased PLT, ultimately resulting in elevated APRI and FIB‐4. However, if acute liver injury is effectively controlled, parenchymal cells can undergo regeneration and replace necrotic or apoptotic cells, leading to gradual decline in these biomarker values. In cases of severe liver damage or persistent hepatic injury, liver regeneration may be impaired, and hepatocytes can be substituted with abundant extracellular matrix, including fibrillar collagen.^[^ ^34^ ^]^ Therefore, early aggressive treatment and regular disease management are crucial for patients with ACLF.

This study has several limitations. First, the study focused on a small proportion of HBV‐ACLF patients from a single region in China, which may restrict its generalizability. Second, while we compared the long‐term mortality between HBV‐ACLF and non‐HBV‐ACLF, while the data of non‐HBV‐ACLF were derived from previously reported studies. Third, the assessment of liver fibrosis relied solely on serum markers, and additional methods such as FibroScan were not employed. Last, the number of decompensation and death events of ACLF survivors in this study was relatively low, which may not fully reflect the overall situation. Therefore, further extensive trials involving larger and more diverse population are needed to validate the long‐term prognosis of patients with HBV‐ACLF.

In conclusion, this prospective longitudinal cohort study further provided evidence that the 90‐day period following the occurrence of HBV‐ACLF was a critical time point for long‐term prognosis, with favorable outcomes observed beyond this timeframe. The study also highlighted that improvement in the general laboratory parameters, such as ALT, AST, TB, INR, AFP, and WBC, and HBV virologic response within one year, whereas changes in fibrosis biomarkers and HBeAg seroconversion required 4–5 years. The high levels of total bilirubin might be the independent risk factor for 8‐year survival of ALCF survivors. Therefore, early and proactive treatment of HBV‐ACLF patients is crucial, and it is equally vital to provide regular and evidence‐based disease management after their discharge from hospital.

## Conflict of Interest

The authors declare no conflict of interest.

## Author Contributions

L.X. and J.C. contributed equally to this work. X.X. and Z.X. provided study concept and design; L.X. and J.C. drafted the manuscript; S.Z., W.Z., K.H., X.Q., and T.L. acquisited data; X.X., Z.X., J.H., J.C., L.X., S.Z., K.H., and T.L. were responsible for collection of the samples; L.X., F.Z., and Q.L. were responsible for analysis and interpretation of data and statistical analysis; X.X. obtained funding; T.L., J.H., D.Z., X.W., and Z.P. provided administrative, technical, and material support; All the authors provided critical revision of the manuscript for important intellectual content.

## Supporting information

Supporting Information

## Data Availability

The data that support the findings of this study are available in the supplementary material of this article.
