# Supplementary material for: The 90‐Day Survival Threshold: A Pivotal Determinant of Long‐Term Prognosis in HBV‐ACLF Patients – Insights from a Prospective Longitudinal Cohort Study
Source: Adv Sci (Weinh). 2024 Feb 21;11(16):2304381. doi: 10.1002/advs.202304381 (PMC11040353; doi:10.1002/advs.202304381)
Supplement: Supplementary file 1 — Supporting Information [file ADVS-11-2304381-s001.pdf]

ADVANCED SCIENCE
Open Access

Supporting Information

for *Adv. Sci.*, DOI 10.1002/advs.202304381

The 90-Day Survival Threshold: A Pivotal Determinant of Long-Term Prognosis in HBV-ACLF Patients – Insights from a Prospective Longitudinal Cohort Study

Lanlan Xiao, Jiajia Chen, Shuai Zhao, Wenxin Zhoudi, Keting He, Xiaohan Qian, Fen Zhang, Qiuhong Liu, Tan Li, Danhua Zhu, Xiaoxin Wu, Zhangya Pu, Jianrong Huang, Zhongyang Xie and Xiaowei Xu**

Supplemental material

Table S1. Follow up schedule

Visiting viewpoint	V1	V2	V3	V4	V5	V6	V7	V8	V9	V10→V25
Follow up time (weeks)	0	1	2	3	4	8	12	24	36	48→408 (24 weeks/per)
Time Window (days)	-14	±2	±2	±2	±3	±7	±7	±7	±7	±7
Sign informed consent										
Basic information										
Inclusion criteria, exclusion criteria										
Past medical history										
Previous medication history										
Vital signs										
Symptoms										
Physical examination										
Complication (Hepatic encephalopathy, hepatorenal syndrome, hemorrhage, infection and hepatopulmonary syndrome)										
ECG										
Lung CT										
Abdomen B-ultrasound										
Blood routine										
Urine routine										
Biochemical test										
Coagulation function										
AFP										
Plasma nitrogen										
Blood gas analysis + lactic acid										
Antinuclear antibody antimitochondrial antibody										
Pregnancy test (Female)										
Virological examination (including HIV)										
Antibodies to hepatitis A, C, D and E virus										
Routine detection of hepatitis B										
HBV-DNA quantification										
Concomitant medication										
MELD score										
Child score										
Condition assessment										
Collect blood										
Adverse event										
Serious adverse events										

Table S2 . Healthy controls and CHB Patients' demographics

Variable	Healthy controls (n=14)	CHB patients (n=24)
Age (years)	25.3 (13.8)	39.7 (8.8)
Male sex (no.)	9 (64.3)	18 (75.0)
ALT (U/L)	22.4 (6.8)	45.4 (60.5)
AST (U/L)	20.5 (6.2)	30.8 (22.2)
Albumin (g/dL)	47.7 (3.6)	45.2 (4.6)
TBIL(μmol/L)	15.2 (4.4)	13.0 (6.5)
Creatinine (μmol/L)	73.6 (12.5)	73.6 (12.5)
Haemoglobin (g/L)	233.3 (120.3)	150.2 (13.3)
Platelet count (10^9/L)	140.0 (19.0)	197.5 (49.8)

Table S3. Baseline clinical and laboratory characteristics

Variable	ACLF survivors beyond 3 months		
	Survived (n=92)	Died or received LT (n=9)	p
Age (years)	40.7 (9.0)	45.5 (7.4)	0.361
Male sex (no.)	77 (83.7)	8 (88.9)	0.684
Liver cirrhosis (no.)	36 (39.1)	4 (44.4)	0.756
HBV-DNA level (IU/mL)			
≤200	2 (2.2)	1 (11.1)	0.132
$200\text{-}2\times10^4$	32 (34.8)	3 (33.3)	0.931
$2\times10^4\text{-}2\times10^6$	37 (40.2)	2 (22.2)	0.29
$\geq2\times10^6$	21 (22.8)	3 (33.3)	0.48
Complications (no.)			
Overt ascites	6 (6.5)	1 (11.1)	0.605
HE	6 (6.5)	1 (11.1)	0.605
Spontaneous bacterial peritonitis	5 (5.4)	1 (11.1)	0.492
Gastrointestinal hemorrhage	5 (5.4)	1 (11.1)	0.492
Infection	8 (8.7)	1 (11.1)	0.808
Hepatorenal syndrome	1 (1.1)	0 (0)	0.753
Laboratory data			
ALT (U/L)	489.6 (575.7)	260.5 (219.8)	0.231
AST (U/L)	293.4 (356.9)	150.5 (96.8)	0.227
Albumin (g/dL)	31.5 (6.8)	29.2 (3.2)	0.366
TBIL(μmol/L)	281.6 (91.6)	378.3 (98.0)	0.696
Creatinine (μmol/L)	62.0 (14.1)	64.5 (15.0)	0.683
White blood cell count (10^9/L)	6.7 (2.9)	5.4 (3.1)	0.532
Neutrophil count (10^9/L)	4.3 (2.4)	3.7 (2.6)	0.636
Haemoglobin (g/L)	132.2 (20.3)	124.5 (21.0)	0.926
Platelet count (10^9/L)	125.1 (57.5)	82.6 (54.0)	0.96
Prothrombin time (s)	22.7 (6.1)	23.6 (7.2)	0.816
INR	1.9 (0.5)	2.0 (0.6)	0.858
Severity score			
MELD	20.3 (3.7)	22.4 (3.2)	0.784

CLIF-C ACLFs	35.1 (5.0)	36.1 (4.9)	0.818
HBV-ACLF (COSSH criteria) (no.)			
pre-ACLF	14 (15.2)	0 (0)	0.207
ACLF grade 1	66 (71.7)	7 (77.8)	0.699
ACLF grade 2	11 (12.0)	2 (22.2)	0.38
ACLF grade 3	1 (1.1)	0 (0)	0.753

**p* <0.05, ***p* <0.01, ****p* <0.001

Table S4. Cox proportional hazards regression model investigating independent risk factors for 8-year survival of ACLF survivors (n=101).

Variable	Reference	HR (CI 95%)	p value
Total bilirubin	Per μmol/L increase	1.008 (1.001-1.015)	0.021

HR, hazard ratio.

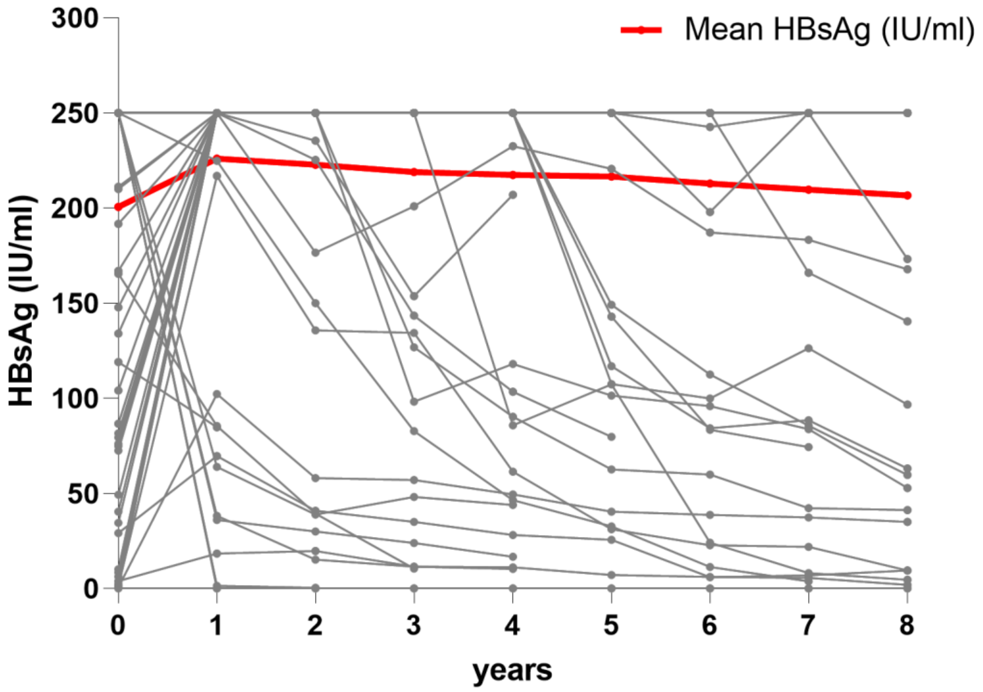

Figure S1. The changes in the level of hepatitis B surface antigen of ACLF survivors (n=101).

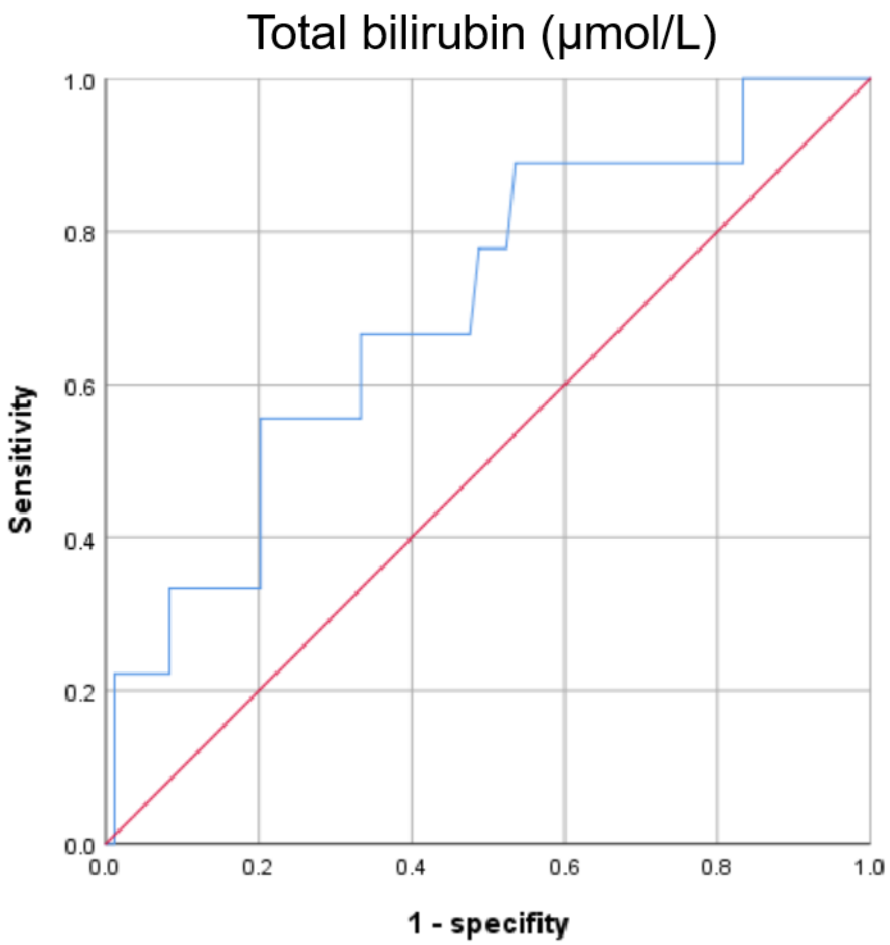

Figure S2. The areas under the receiver operating curve for total bilirubin for 8-year survival of ALCF survivors (n=101).

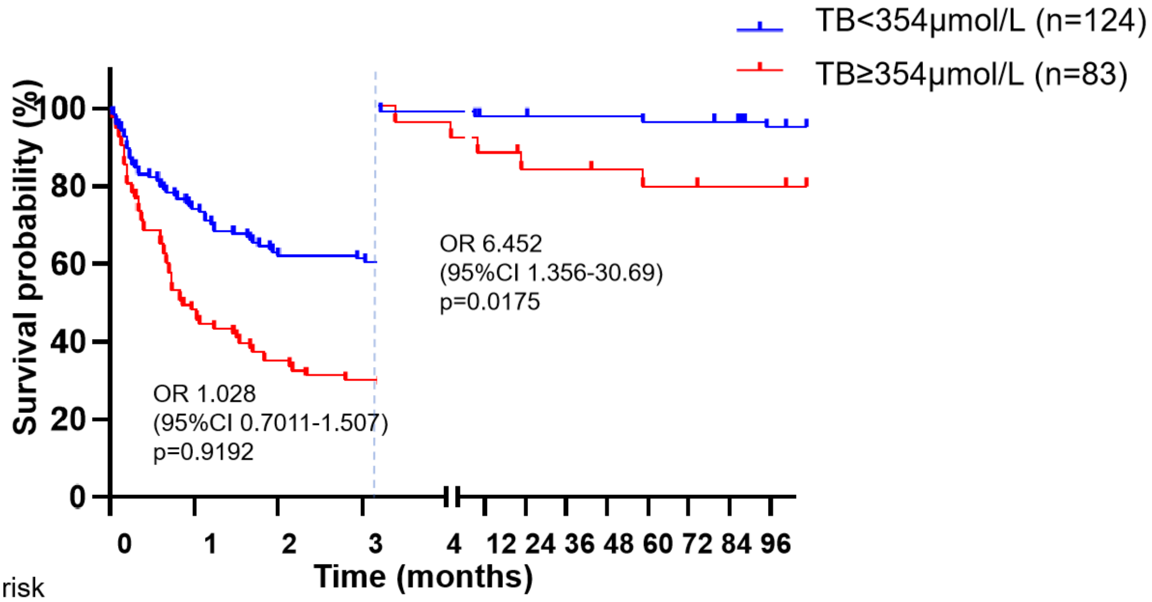

No. at risk												
Overall	207	132	106	101	95	92	92	91	89	88	87	84
TB<354μmol/L	124	92	77	76	73	72	72	72	71	70	70	70
TB≥354μmol/L	83	40	29	25	22	20	20	19	18	18	17	14

Figure S3. Landmark analysis for ACLF survivors based on the cut off value of Total bilirubin levels (n=207).

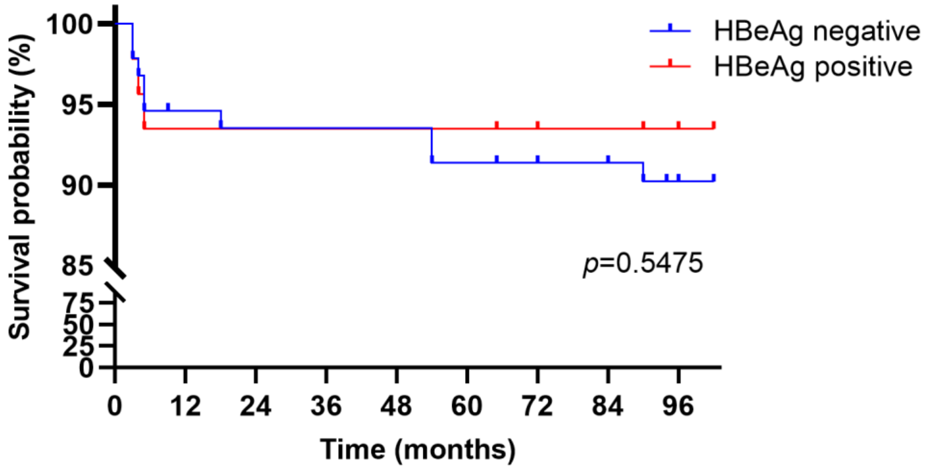

Figure S4. Long-term survival curves for ACLF survivors based on whether HBeAg was positive or not (n=101).
